# Anticoagulant rodenticide blood‐clotting dose‐responses and resistance factors for Tyrosine139Cysteine (Y139C) heterozygous‐ and homozygous‐resistant house mice (*Mus musculus*)

**DOI:** 10.1002/ps.7066

**Published:** 2022-08-18

**Authors:** Mhairi Alyson Baxter, Alan Peter Buckle, Stefan Endepols, Colin Vittorio Prescott

**Affiliations:** ^1^ School of Biological Sciences, Health and Life Sciences Building The University of Reading Reading UK; ^2^ Bayer AG CropScience Research and Development Public Health Monheim Germany

**Keywords:** Anticoagulant resistance, house mouse, Tyrosine139Cysteine, Y139C, blood clotting response test, resistance factor

## Abstract

**Background:**

The house mouse (*Mus musculus*) is a globally distributed rodent pest species against which anticoagulant rodenticides are widely used for the protection of human and animal health and the conservation of threatened wildlife. Anticoagulant‐resistant house mice have been known for more than half a century. A house mouse strain was developed in the laboratory that was homozygous resistant for the single nucleotide polymorphism (SNP) Tyrosine139Cysteine (Y139C) and, subsequently, heterozygous resistant animals were produced from this strain by crossing with the homozygous susceptible strain.

**Results:**

Using blood clotting response tests, resistance factors at the ED_50_ level in the homozygous resistant strain for the first‐generation anticoagulants warfarin, chlorophacinone, diphacinone and coumatetralyl were in the range 31.5 to 628.0 for males (M) and 21.6 to 628.0 for females (F), thus indicating that Y139C house mice are substantially resistant to all these substances. Resistance factors at the ED_50_ level for the homozygous strain generated against the second‐generation compounds were: brodifacoum (M, 1.7; F, 1.9), bromadiolone (M, 16.6; F, 21.0), difenacoum (M, 1.2; F, 2.7), difethialone (M, 1.5; F, 1.5), and flocoumafen (M, 0.9; F, 1.2). Equivalent values for the heterozygous strain were: brodifacoum (M, 1.6; F, 1.4), bromadiolone (M, 5.6; F, 6.5), difenacoum (M, 1.0; F, 1.3), difethialone (M, 1.1; F, 1.1), flocoumafen (M, 0.9; F, 1.1).

**Conclusion:**

Y139C SNP homozygous resistant mice are more resistant to anticoagulants than heterozygous resistant animals. All first‐generation anticoagulants are highly resisted and, among the second‐generation compounds, Y139C mice are resistant to bromadiolone and sometimes to difenacoum. © 2022 The Authors. *Pest Management Science* published by John Wiley & Sons Ltd on behalf of Society of Chemical Industry.

## Graphical abstract

Resistance factors for Y139C homozygous resistant *Mus musculus* are given for first‐generation anticoagulants (FGAR) chlorophacinone, coumatetralyl, diphacinone and warfarin sodium and for both homozygous and heterozygous strains against second‐generation anticoagulants (SGAR) brodifacoum, bromadiolone, difenacoum, difethialone and flocoumafen. There is substantial resistance to all FGARs and to bromadiolone among the SGARs.

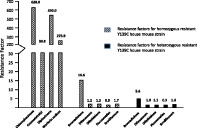

## 1 INTRODUCTION

The house mouse (*Mus musculus*) is a ubiquitous commensal rodent pest and the depredations of this species, in terms of the damage it inflicts and diseases it transmits, are well known.^1^, ^2^ Anticoagulant rodenticides are applied almost universally to combat house mouse infestations because they are effective,^3^ and when applied using all available and appropriate risk mitigation measures, and used against house mice generally indoors, carry limited risk to non‐target animals and humans.^4^, ^5^, ^6^ However, this species displays a fundamental difference to the other common commensal rodent pest, the Norway rat (*Rattus norvegicus*), in its relatively low intrinsic susceptibility to anticoagulants.^3^ This characteristic, sometimes called ‘natural resistance’,^7^, ^8^ was demonstrated in early work with the active substance warfarin. To obtain 99% mortality in Norway rats, a period of 5.8 days of continuous no‐choice feeding on 50 ppm warfarin bait was required.^3^ To obtain the same degree of mortality in house mice, 29.5 days of continuous feeding on 250 ppm warfarin was needed. However, in spite of the relative insensitivity of house mice to warfarin, and probably to other first‐generation anticoagulants (FGARs), these substances were widely and effectively used against them until the advent of resistance.^3^ The mechanism of natural resistance is uncertain but is likely to involve either differences in the physiology of the vitamin K cycle, the blood clotting mechanism, or differences in the efficiency of mechanisms for the elimination of toxins, such as P‐450 cytochrome systems, or a combination of both.^8^ In addition, and again in comparison to Norway rats, house mice also eat from a wider variety of food sources, which has the effect of reducing the intake of rodenticide baits.^9^

Single nucleotide polymorphisms (SNP) that confer resistance to anticoagulant rodenticides are also common in house mice,^10^ although Endepols et al. found control problems with mouse infestations that could not be attributed to known resistance SNPs.^11^ Recent surveys in Germany, Ireland, the UK and France have shown resistance SNPs at a high prevalence among house mouse populations.^12^, ^13^, ^14^, ^15^ Two SNPs, Leuceine128Serine (L128S) and Tyrosine139Cysteine (Y139C), are particularly common and a third linked group of sequence changes, Arginine12Tryptophan /Alanine26Serine/Alanine48Threonine/Arginine61Leucine (also known as ‘*spretus*’ introgression) is also found in some countries.^10^, ^12^, ^13^, ^17^ Maps showing the known occurrence of these SNPs in wild house mouse populations are published on‐line by the Rodenticide Resistance Action Committee (RRAC) of CropLife International (see www.rrac.info).

A question that arises immediately from this is how do these SNPs affect the efficacy of anticoagulant rodenticide applications to control mouse infestations that carry them? A metric that is commonly employed to quantify the influence of resistance on practical control is the ‘resistance factor’ (sometimes known as ‘resistance ratio’).^10^ This is defined as the multiple of the dose required against a susceptible strain to obtain the same effect against a resistant strain.^18^ Resistance factors are usually calculated using doses measured at the 50^th^ percentile of the effective or lethal dose (i.e. ED_50_ or LD_50_), from either acute oral toxicity or blood‐clotting response (BCR) tests.^19^, ^20^, ^21^ A significant advantage of BCR testing is that the procedure permits the adoption of higher standards of animal welfare than earlier acute oral toxicity and feeding tests, which relied on a severe end‐point, namely death.

Resistance factors are useful in the management of resistant rodent infestations because they permit the relative levels of resistance to active substances to be compared. Active substances with lower resistance factors against a resistant rodent strain will be more effective than those with higher factors. For any active ingredient, an impact on control in the field (practical resistance)^10^ will occur when the resistance factor is greater than a particular threshold, which in turn will be dependent both on the bait formulation strength and the LD_50_ of the active ingredient against the target susceptible strain. It is usually considered that resistance factors less than three to five, depending on the compound, are unlikely to have a significant detrimental effect on the performance of an active substance, factors in the range five to ten may have some effect and factors greater than ten are very likely to impair the satisfactory control of infestations that possess them.^10^

This paper describes laboratory work, using BCR tests, to establish the susceptibility of the homozygous strain of Y139C resistant house mice to the FGARs warfarin sodium, chlorophacinone, coumatetralyl and diphacinone and the second‐generation anticoagulant rodenticides (SGAR) brodifacoum, bromadiolone, difenacoum, difethialone and flocoumafen. The response of the heterozygous Y139C strain to the five SGARs was also established. A necessary first step in the establishment of resistance factors are data on the fully susceptible strain. Prescott et al. provided this baseline information for susceptible house mice using BCR tests for the five SGARs, permitting resistance factors to be determined in the work described here for Y139C mice for both homozygous and heterozygous strains.^21^ This is the first time that different resistance factors have been reported for homozygous and heterozygous resistance house mice.

## 2 MATERIALS AND METHODS

### 2.1 Background

Prescott et al. examined the effects of variations in different BCR testing systems and proposed a standardized procedure for the establishment of susceptibility baselines and for subsequent testing of resistant strains.^21^ Experimental procedures used throughout the present study are as described by those authors. Further explanation of the rationale for the methods adopted and their details is provided by Pelz and Prescott.^10^

### 2.2 Animals ‐ Anticoagulant susceptible strain

The susceptible albino house mouse strain was an outbred CD‐1 strain of house mouse supplied by Charles River UK Ltd. All mice were healthy, active, sexually mature and at least 4 weeks old. After arrival, they were held for a settling‐in period of at least 3 days before testing. During this period and the period between dose administration and blood sampling they were provided with Special Diet Services Rat and Mouse No. 3 pelleted diet (https://sdsdiets.com/wp-content/uploads/2021/02/rm3-e-fg.pdf) and water *ad libitum*.

### 2.3 Animals ‐ Anticoagulant resistant strains

In the 1980s, a population of wild house mice trapped from locations around Reading, UK, was found to be ‘warfarin resistant’.^22^ Wild‐caught individuals held at the University of Reading were fed 250ppm warfarin in a lethal feeding period test of 21 days then maintained for 28 days observation to determine their ‘warfarin resistance’, and 80% of the mice survived. Thirty individuals derived from the breeding stocks subsequently established were dosed with bromadiolone via oral gavage, and among thirty animals dosed three (10%) died.^22^ This level of resistance to both warfarin and bromadiolone suggested a degree of cross‐resistance in this strain of mice.^10^

Breeding studies were established to examine the inheritance of bromadiolone resistance. Wild‐caught individuals of unknown genotype were crossed with susceptible Swiss house mice and the progeny tested for resistance using a test dose of bromadiolone (10 mg kg^‐1^ b.w.) that would be lethal to susceptible individuals. Assuming the unifactorial dominant inheritance of resistance, it was expected that some wild‐caught individuals would be homozygous resistant (RR), some heterozygous resistant (Rr) and some susceptible (rr),^23^ and in test crosses 100% of the progeny of homozygous resistant parents, and 50% of the progeny of heterozygous resistant parents would be expected to survive the test dose of bromadiolone. The resistance gene was transferred over six generations onto the anticoagulant susceptible strain (outbred CD‐1 strain obtained from Charles River UK Ltd), and a breeding nucleus of house mice homozygous for the VKORC1 mutation Y139C was subsequently created from individuals identified as homozygous resistant in these breeding studies.^22^ The strain was maintained at the University of Reading and the SNP it carried was subsequently identified as Y139C.^24^

Heterozygous animals used in this study were generated by crossing male house mice homozygous for the VKORC1 mutation Y139C (as described above) with susceptible albino female CD‐1 house mice, thus producing an F1 progeny heterozygous for the VKORC1 mutation Y139C.

### 2.4 Test Substances

Concentrates for the four FGAR substances, chlorophacinone, diphacinone, coumatetralyl and warfarin sodium, and five SGAR substances, brodifacoum, bromadiolone, difenacoum, difethialone and flocoumafen were obtained either directly from manufacturers or from chemical supply houses. The isomer ratios of the samples provided were not known but presumed to be similar to those normally found in proprietary bait formulations. Each sample was stored and identified using a unique reference number. Concentrates were diluted using polyethylene glycol (PEG) 200 to obtain the required concentrations in order to deliver measured doses of the active substances depending on the weight of each test animal.^21^

### 2.5 BCR Resistance Tests

Animals were caged individually or in groups and provided with food and water *ad libitum*. Ambient conditions in the animal rooms were maintained within limits set by best practice guidance (i.e. Universities Federation for Animal Welfare); namely a temperature range of 18‐24^o^C, relative humidity within the range 40%‐70%, between 10 and 25 air changes an hour and a 12 h light‐dark cycle. Individual animals were identified by cage label and group caged animals by tail marks.

Caged animals were weighed to an accuracy of 0.1 g prior to dosing with anticoagulant. Anticoagulant, at the required concentration, was delivered to the test animal by oral gavage at the rate of 1.0 mL per 100 g body weight. The concentration delivered varied depending on the active substance under investigation and the intended dose rate.

After dosing, test animals were again provided with food and water *ad libitum*, and after 24‐hours a blood sample (0.9 ml) was taken under terminal isoflurane anesthesia using 3.2% sodium citrate (0.1 ml) as the anticoagulant. Plasma was obtained by centrifugation and clotting time determined in triplicate using Diagen rabbit brain thromboplastin and an Amelung KC4 micro semi‐automatic hemostasis instrument.^21^

Test animals were considered to be responders when their coagulation time was equivalent to an International Normalised Ration (INR) ≥5.^21^ A coagulation time of 47.5 seconds was equivalent to an INR of 5 when using Diagen rabbit brain thromboplastin. Group size was increased, and further dosage levels selected to generate dose‐response data with acceptable fiducial limits using probit analysis, while making every effort to use the minimum possible number of animals.

### 2.6 Generation of dose‐response data

Prescott et Al. provided dose‐response data for the susceptible mouse strain for the five SGAR active substances.^21^ In the present study, dose‐response data were generated for the four FGARs using the susceptible strain, for both FGARs and SGARs using the homozygous resistant strain and for SGARs using heterozygous resistant animals.

Probit analysis was used to examine the dose‐response data.^25^ Using the SAS System for Windows, Version 8.02, the Proc GENMOD was used to determine whether the probit lines for the two sexes were coincident, parallel or separate. Output from Proc GENMOD produced deviance values for the three assumptions and ‘Chi Square’ was used to determine whether the differences were significant.^21^Subsequently, probit dose‐response data for each sex was generated using the Proc PROBIT taking results of Proc GENMOD into account.^21^ Probit analysis was used to analyze dose‐response data and to generate dose‐response percentiles for each active ingredient and sex combination for both the homozygous and heterozygous resistant mice. Statistical differences in the responses of the two sexes were tested and analysis of data was carried out three times for each substance, assuming the probit response of the two sexes were either coincident, parallel or separate.^21^ Resistance factors were determined, at the ED_50_ level, as the multiple of the dose administered to a susceptible strain required to obtain the same effect on blood clotting in the resistant strain.^10^ In a similar way, the Proc GENMOD was used to determine whether the probit lines for the homozygous and heterozygous resistant animals of each sex deviated significantly from coincidence.

## 3 RESULTS

### 3.1 Resistance baselines and resistance factors for four first generation anticoagulants

An initial requirement in the study of resistance in house mice to FGAR active substances by BCR testing was the establishment of susceptibility baselines for the four active substances.^10^, ^21^ FGAR doses administered, numbers of animals dosed and numbers of responders in experiments to generate these baselines are given as supplementary information (Table S1). Using Proc GENMOD, probit lines were found to be coincident for the two sexes for chlorophacinone, diphacinone and warfarin sodium (i.e. not deviating significantly from a parallel or a coincident response) and parallel for coumatetralyl (i.e. not deviating significantly from a parallel response but deviating significantly from a coincident response). Using Proc PROBIT, and taking into account the above output from Proc GENMOD, dose‐response data for each of the four FGAR's against the susceptible strain were generated and are summarized in Table 1.

**Table 1:** Summary probit dose‐response data for male and female susceptible and homozygous Y139C resistant house mice for four FGAR active ingredients. Dose‐response data were insufficient to generate fiducial limits for some active substances

		Mean effective dose (mg kg^‐1^) with lower and upper 95% fiducial limits			
		Susceptible house mouse			
Sex	Effective Dose (%)	Chlorophacinone	Coumatetralyl	Diphacinone	Warfarin sodium
Male	1	0.34 (0.11‐0.49)	0.29 (0.04‐0.58)	0.53 (0.07‐0.68)	0.42 (0.11‐0.68)
	40	0.78 (0.59‐0.90)	1.59 (1.03‐2.02)	0.86 (0.66‐1.07)	1.30 (0.89‐1.68)
	50	0.86 (0.70‐1.01)	1.95 (1.43‐2.51)	0.91 (0.76‐1.29)	1.50 (1.10‐1.96)
	60	0.94 (0.80‐1.16)	2.40 (1.87‐3.30)	0.97 (0.83‐1.64)	1.72 (1.32‐2.35)
	99	2.15 (1.56‐5.76)	13.04 (7.00‐72.90)	1.57 (1.18‐17.02)	5.41 (3.46‐17.54)
Female	1	0.34 (0.11‐0.49)	1.55 (0.77‐2.20)	0.53 (0.07‐0.68)	0.42 (0.11‐0.68)
	40	0.78 (0.59‐0.90)	4.41 (3.56‐5.17)	0.86 (0.66‐1.07)	1.30 (0.89‐1.68)
	50	0.86 (0.70‐1.01)	5.02 (4.18‐5.89)	0.91 (0.76‐1.29)	1.50 (1.10‐1.96)
	60	0.94 (0.80‐1.16)	5.70 (4.85‐6.80)	0.97 (0.83‐1.64)	1.72 (1.32‐2.35)
	99	2.15 (1.56‐5.76)	16.24 (11.84‐30.26)	1.57 (1.18‐17.02)	5.41 (3.46‐17.54)
		Homozygous resistant house mice			
Male	1	302.38	1.61 (0.00‐6.39)	283.38	96.13
	40	506.76	41.37 (20.40‐62.77)	461.84	351.73
	50	539.78	61.50 (40.10‐119.49)	490.24	412.16
	60	574.94	91.43 (60.63‐295.76)	520.4	482.97
	99	963.56	2345 (517.38‐1697868)	848.11	1767.00
Female	1	302.38	83.19	283.38	186.68
	40	506.76	104.8	461.84	683.08
	50	539.78	107.8	490.24	800.43
	60	574.94	110.89	520.4	937.94
	99	963.56	139.7	848.11	3432.00

Following the establishment of these baselines, the response of the homozygous resistant strain was evaluated for the four FGAR active substances using the same BCR methodology (Table 1 and Table S2). However, the high degree of resistance in this strain to the FGARs resulted in dose‐response data that, when analyzed using Proc PROBIT, was not sufficient to generate fiducial limits for chlorophacinone, diphacinone or warfarin sodium against both sexes, and for coumatetralyl against female mice (Table 1). Operational constraints and ethical considerations limited the numbers of doses that could be tested, with doses up to 500 mg.kg^‐1^ producing responders (with prolonged clotting times) in less than 50% of animals tested. Thus, with the exception of coumatetralyl data against male mice, resistance factors presented in Table 2, generated from ED data presented in Table 1, should be considered with a degree of caution. However, in all cases, the resistance factors were very high and signify that the FGAR substances would be unlikely to be effective in the control of Y139C resistant house mice.

**Table 2:** Calculated resistance factors (RF) at the ED_50_ and ED_99_ for male and female house mice homozygous for the VKORC1 mutation Y139C against four FGAR substances. The RF for male mice against coumatetralyl may be considered precise. However, RFs for females and for males against the other three test substances should be considered indicative

Sex	Effective dose (%)	Active substance			
		Chlorophacinone	Coumatetralyl	Diphacinone	Warfarin sodium
male	50	628.0	30.8	538.0	275.0
	99	448.2	179.8	540.2	326.6
female	50	628.0	21.6	538.0	533.0
	99	448.2	8.6	540.2	634.4

### 3.2 Resistance baselines and resistance factors for the five second generation anticoagulants against homozygous and heterozygous Y139C resistant house mice

Varying doses of the five SGARs were administered to homozygous and heterozygous resistant house mice, and their BCR responses were observed as described above (Table S3) and dose‐responses were analyzed using probit analysis (Table 3). As before for the FGARs, Proc GENMOD was used to compare the dose‐responses of male and female mice to the five SGAR active substances. For the homozygous Y139C resistant mice, probit lines of the two sexes were found to be separate for bromadiolone and flocoumafen, parallel for difenacoum and coincident for brodifacoum and difethialone. For the heterozygous mice, the probit lines of the two sexes were found to be separate for brodifacoum, parallel for difenacoum and coincident for bromadiolone, difethialone and flocoumafen. Dose‐response data for the susceptible mouse strain were already available for brodifacoum, bromadiolone, difenacoum, difethialone and flocoumafen.^21^ Resistance Factors were calculated accordingly for homozygous and heterozygous animals of the Y139C resistant strain (Table 4).

**Table 3:** Summary probit dose‐response data for male and female susceptible, homozygous and heterozygous Y139C resistant house mice for the five active SGAR active substances. Data for the susceptible strain are reproduced from Prescott *et al*.^21^

		Mean effective dose (mg kg^‐1^) with lower and upper 95% fiducial limits				
		Susceptible house mouse*				
Sex	Effective Dose (%)	Bromadiolone	Difenacoum	Difethialone	Flocoumafen	Brodifacoum
Male	40	1.89 (1.75–2.00)	0.81 (0.72–0.88)	0.78 (0.71–0.82)	0.49 (0.45–0.52)	0.38 (0.35–0.39)
	50	1.96 (1.84–2.09)	0.85 (0.76–0.92)	0.83 (0.77–0.87)	0.51 (0.47–0.55)	0.39 (0.37–0.40)
	60	2.03 (1.91–2.19)	0.89 (0.80–0.97)	0.88 (0.82–0.93)	0.53 (0.49–0.57)	0.40 (0.38–0.41)
	99	2.72 (2.42–3.58)	1.27 (1.12–1.61)	1.46 (1.28–1.83)	0.74 (0.65–0.96)	0.51 (0.47–0.57)
Female	40	1.66 (1.62–1.70)	0.54 (0.49–0.58)	0.78 (0.71–0.82)	0.42 (0.37–0.45)	0.34 (0.32–0.35)
	50	1.68 (1.64–1.73)	0.56 (0.52–0.60)	0.83 (0.77–0.87)	0.44 (0.39–0.47)	0.35 (0.33–0.36)
	60	1.70 (1.66–1.76)	0.59 (0.54–0.64)	0.88 (0.82–0.93)	0.45 (0.41–0.49)	0.36 (0.34–0.37)
	99	1.87 (1.79–2.06)	0.84 (0.74–1.07)	1.46 (1.28–1.83)	0.63 (0.56–0.81)	0.46 (0.42–0.51)
		Homozygous resistant				
Male	1	1.70 (0.06‐5.23)	0.52 (0.31‐0.65)	0.30 (0.13‐0.43)	0.09 (0.01‐0.17)	0.31 (0.18‐0.40)
	40	23.59 (10.70‐34.18)	0.97 (0.84‐1.07)	1.09 (0.92‐1.44)	0.37 (0.21‐0.46)	0.63 (0.56‐0.68)
	50	32.54 (18.75‐46.21)	1.05 (0.94‐1.15)	1.27 (1.06‐1.85)	0.44 (0.32‐0.56)	0.68 (0.62‐0.75)
	60	44.88 (30.21‐67.93)	1.13 (1.03‐1.26)	1.49 (1.19‐2.41)	0.53 (0.42‐0.75)	0.74 (0.69‐0.84)
	99	623.13 (244.39‐9726)	2.13 (1.73‐3.40)	5.34 (3.00‐22.96)	2.32 (1.25‐29.62)	1.49 (1.18‐2.62)
Female	1	16.43 (8.15‐21.49)	0.74 (0.47‐0.90)	0.30 (0.13‐0.43)	0.32 (0.23‐0.37)	0.31 (0.18‐0.40)
	40	32.49 (26.93‐37.76)	1.38 (1.25‐1.53)	1.09 (0.92‐1.44)	0.48 (0.45‐0.52)	0.63 (0.56‐0.68)
	50	35.31 (30.11‐41.87)	1.49 (1.36‐1.68)	1.27 (1.06‐1.85)	0.51 (0.48‐0.56)	0.68 (0.62‐0.75)
	60	38.38 (33.16‐47.15)	1.61 (1.47‐1.87)	1.49 (1.19‐2.41)	0.54 (0.50‐0.60)	0.74 (0.69‐0.84)
	99	75.88 (57.36‐158.24)	3.03 (2.40‐5.18)	5.34 (3.00‐22.96)	0.81 (0.69‐1.21)	1.49 (1.18‐2.62)
		Heterozygous resistant				
Male	1	4.50 (2.70‐6.00)	0.75 (0.68‐0.79)	0.32 (0.16‐0.44)	0.36 (0.32‐0.39)	0.24 (0.10‐0.34)
	40	9.90 (8.10‐11.50)	0.87 (0.84‐0.91)	0.79 (0.63‐0.92)	0.46 (0.44‐0.48)	0.56 (0.42‐0.67)
	50	10.90 (9.20‐12.60)	0.89 (0.86‐0.93)	0.89 (0.74‐1.02)	0.47 (0.46‐0.50)	0.62 (0.49‐0.74)
	60	12.00 (10.30‐13.90)	0.91 (0.88‐0.95)	1.0 (0.85‐1.10)	0.49 (0.47‐0.51)	0.69 (0.57‐0.83)
	99	26.40 (21.30‐38.60)	1.10 (1.0‐1.2)	2.50 (2.0‐4.1)	0.62 (0.58‐0.70)	1.64 (1.24‐3.11)
Female	1	4.50 (2.70‐6.00)	0.61 (0.56‐0.65)	0.32 (0.16‐0.44)	0.36 (0.32‐0.39)	0.38 (0.31‐0.42)
	40	9.90 (8.10‐11.50)	0.72 (0.69‐0.74)	0.79 (0.63‐0.92)	0.46 (0.44‐0.48)	0.48 (0.45‐0.52)
	50	10.90 (9.20‐12.60)	0.73 (0.71‐0.75)	0.89 (0.74‐1.0)	0.47 (0.46‐0.50)	0.50 (0.46‐0.54)
	60	12.00 (10.30‐13.90)	0.75 (0.73‐0.77)	1.00 (0.85‐1.10)	0.49 (0.47‐0.51)	0.51 (0.48‐0.56)
	99	26.40 (21.30‐38.60)	0.88 (0.83‐0.96)	2.50 (2.00‐4.10)	0.62 (0.58‐0.70)	0.65 (0.59‐0.84)

**Table 4:** Calculated resistance factors (RF) at ED_50_ and ED_99_ for male and female house mice either heterozygous or homozygous for the VKORC1 mutation Y139C against the five SGAR active substances

Strain	Sex	Effective dose (%)	Active substance				
			Bromadiolone	Difenacoum	Difethialone	Flocoumafen	Brodifacoum
Homozygous resistant	male	50	16.6	1.2	1.5	0.9	1.7
		99	229.1	1.7	3.7	3.1	2.9
	female	50	21.0	2.7	1.5	1.2	1.9
		99	40.6	3.6	3.7	1.3	3.2
Heterozygous resistant	male	50	5.6	1.0	1.1	0.9	1.6
		99	9.7	0.9	1.7	0.8	3.2
	female	50	6.5	1.3	1.1	1.1	1.4
		99	14.1	1.0	1.7	1.0	1.4

The ED_50_ data show that both homozygous resistant male and female mice are more tolerant of the five SGARs than are heterozygous resistant individuals (Table 3). The response of homozygous resistant mice was compared with that of heterozygous mice for each active substance and sex combination using SAS Probit (Proc GENMOD) (see Table 5). For difenacoum and flocoumafen against both sexes, and for brodifacoum against females and bromadiolone against males, the probit dose‐response generated separate lines for homozygous and heterozygous resistant mice that deviated significantly from parallel lines (with p values ranging between <0.001 and <0.02). For bromadiolone and difethialone against females, the probit dose‐response generated parallel lines for homozygous and heterozygous resistant mice that deviated significantly from coincidence (p < 0.001 and p<0.005 respectively). For brodifacoum and difethialone against male animals, the probit dose response generated coincident lines for homozygous and heterozygous resistant mice. The probit dose‐response for the heterozygous resistant mice differed significantly from that of the homozygous resistant strain for all five active ingredients against female animals, and for bromadiolone, difenacoum and flocoumafen against male animals. The inability to demonstrate significance in a similar way for difethialone and brodifacoum against male animals was probably the result of small sample sizes. For ethical reasons, our experiments were conducted using the minimum number of animals to produce probit dose‐responses with fiducial limits.

**Table 5:** Statistical analysis of the data shown in Table 3 providing a comparison of probit dose‐reponse lines for male and female house mice that were either homozygous or heterozygous for the VKORC1 resistance mutation Y139C (*p* values in bold font are statistically significant at the 95% probability level)

Active substance	Sex	Deviance Separate	Deviance Parallel	Deviance Coincident	χ^2^ – from parallel	*p*‐value	χ^2^ – from coincident	*p*‐value
Brodifacoum	F	3.53	9.50	22.89	5.98	**<0.02**	13.39	<0.001
	M	4.59	4.64	5.20	0.06	0.815	0.55	0.457
Bromadiolone	F	1.74	2.68	51.90	0.93	0.334	49.22	**<0.001**
	M	6.34	16.26	48.00	9.92	**<0.002**	31.74	<0.001
Difenacoum	F	7.46	15.39	52.19	7.93	**<0.005**	36.80	<0.001
	M	5.48	26.26	32.10	20.78	**<0.001**	5.84	<0.02
Difethialone	F	12.17	12.31	22.52	0.15	0.700	10.21	**<0.005**
	M	6.50	8.91	9.50	2.40	0.121	0.59	0.443
Flocoumafen	F	10.52	16.58	19.44	6.05	**<0.02**	2.86	0.091
	M	13.11	37.04	37.22	23.94	**<0.001**	0.18	0.674

## 4 DISCUSSION AND CONCLUSIONS

Resistance to the anticoagulant rodenticides in house mice has been known for more than half a century.^7^, ^10^, ^27^ Indeed, early work questioned whether house mice were sufficiently susceptible to warfarin to permit the effective use of the compound against them.^28^ This was due to a combination of low inherent susceptibility and the behavior of house mice which feed sporadically from many different food sources, sometimes preventing the continuous uptake of poisoned bait required for the effectiveness of this and other FGARs. However, our understanding of anticoagulant resistance in house mice has lagged behind that of the other common commensal pest, *Rattus norvegicus*, both in terms of the number of studies conducted and the scope of scientific investigations.^8^, ^29^ Several complicating factors hamper our ability to study and understand house mouse resistance more comprehensively including: 1) the level of natural resistance already mentioned,^7^, ^8^ which is often confused with acquired resistance caused by genetic mutation, 2) that resistance in this species may be the outcome of more than one resistance mechanism^10^ and, 3) the fact that house mice are of lower priority as research subjects because they are considered to be of lesser importance as commensal pests and in disease transmission.^8^, ^29^, ^30^

Many different techniques are employed to study anticoagulant resistance; all have advantages and disadvantages. The ‘gold standard’ resistance test is where candidate rodenticides are applied against field populations of known resistance status.^10^, ^24^ These tests, favoured by those who pioneered the study of house mouse anticoagulant resistance,^31^, ^32^ provide ‘real‐world’ evidence of efficacy against rodents behaving naturally and with alternative foods available. However, they are now rarely conducted because they are time‐consuming and expensive. In their place, researchers now carry out a range of laboratory evaluations including simple feeding studies,^20^, ^33^, ^34^ in which rodents are fed measured doses of anticoagulant baits, and studies of the genetics of individual animals and populations.^12^, ^23^ All of these tests have value and provide different information about anticoagulant resistance. Indeed, to obtain a full understanding of the nature of resistance and its practical consequences, all have a part to play.

The technique of blood clotting response (BCR) tests has a long history in the study of anticoagulant resistance and has several important advantages.^10^, ^21^, ^33^, ^35^, ^36^ Tests are conducted in the laboratory and therefore conditions are largely under the control of the experimentalist. They do not depend on food consumption by confined animals over long periods, as do some feeding tests. The active substance is accurately administered by gavage, and tests are normally completed within 24 hours of dosing. The end‐point in BCR tests is obtained before the test subjects show any acute symptoms of anticoagulant toxicosis and the tests have relatively low adverse impacts on animal welfare. The strength of BCR testing is that when applied across a range of active substances, as in this study, it permits quantitative comparisons between them using Resistance Factors. It does not, however, provide absolute evidence of the practical impacts of different resistances; additional information from other laboratory and field studies is required for that purpose.^26^

Two single nucleotide polymorphisms (SNPs), L128S and Y139C, are predominant markers of resistance in house mice in many countries.^10^ A third important mutation, or rather series of linked mutations, termed the *spretus* introgression, is found in several European countries.^12^ Where they occur, these SNPs are frequently very prevalent in house mouse populations.^12^, ^13^, ^14^, ^15^, ^16^, ^20^

The results of BCR tests on the four FGARs against homozygous Y139C resistant mice that we report here, with resistance factors at the ED_50_ level in the range 21.6 to 628, show that none of the four substances is likely to be effective against Y139C‐resistant mice. The widespread nature of Y139C house mouse resistance and these very high resistance factors support the recommendation that none of these substances should be used against house mice in Europe.^15^, ^37^ These results are very likely applicable wherever the Y139C resistance strain is present and suggest that FGARs should be used for house mouse control only when it, and other resistance mutations, are known to be absent.^10^, ^27^, ^37^

The results for the SGARs against the homozygous strain are apparently equally conclusive. Only the RFs for bromadiolone (16.3 and 20.8, for males and females respectively at the ED_50_ level) appear to be large enough to suggest that there may be a practical level of resistance to that substance.^18^ This supports the recommendation that bromadiolone should not be used against Y139C resistance house mice.^37^ There is no scientific evidence of practical resistance in house mice to brodifacoum, difethialone and flocoumafen, and the work described here and the early field trials of Rowe and his co‐workers, show that these substances may be expected to be fully effective against homozygous resistant Y139C house mice. When these substances fail to be completely effective, either an additional and probably metabolic mode of resistance must be suspected or there may be behavioural causes. The situation with difenacoum appears to be less certain however. Although RFs for this substance reported here are generally quite low, it is apparent from early work that there were mice within some populations in the UK that were fully resistant to difenacoum more than four decades ago.^32^ It may also be that a resistance factor for difenacoum and female homozygous house mice of 2.7 at the ED_50_ level (Table 4) may be sufficient to result in control failures, especially when bait consumption is limited by abundant alternative foods. As mentioned earlier, resistance factors in themselves only provide an indication of the likely impact of resistance on field efficacy. It is also important to consider the intrinsic toxicity of the active substance involved and the concentration in which it is used in product formulations, because both influence the quantity of bait needed to deliver a lethal dose. Hence, a resistance factor of five may not result in control failure when a highly potent active substance is used that requires the consumption of a only few grammes of bait to deliver a lethal dose; whereas the same resistance factor or even a smaller one, occurring with a much less potent substance, may result in a significant diminution in treatment efficacy.

We report here for the first time the results of BCR resistance tests on heterozygous resistant house mice. For two of the SGAR substances, brodifacoum and flocoumafen, effective doses at the ED_50_ level are approximately similar for heterozygous and homozygous house mice (Table 3) and therefore the RFs do not differ appreciably (Table 4). However, effective doses for the other three SGARs are between 1.5 to 3 times higher for the homozygous strain, with concomitant effects on the RFs. It is unsurprising that we find effective doses and RFs for the heterozygous strain lower than those of the homozygous strain, as a similar relationship was observed between RFs for homozygous and heterozygous Y139F‐resistant Norway rats.^38^

Rodent management using rodenticides exerts genetical selection pressure for physiological resistance and also, probably, certain behavioral foraging types.^10^, ^39^ Historically the assessment of resistance in the laboratory was restricted to the identification of animals that were either resistant or not.^7^, ^22^, ^27^ However, with the development of the molecular resistance tests it became possible to differentiate between homozygous resistance and heterozygous resistance,^12^, ^24^ and it quickly became evident in certain regions of the UK with a very high incidence of resistance that the majority of animals were homozygous for a particular mutation, for example house mice with either the L128S or Y139C VKORC1 sequence variants in the London area; and Norway rats with the L120Q VKORC1 sequence variant in central southern England.^15^ Data generated to date strongly suggests that where anticoagulants are not completely effective, they will selectively control susceptible and heterozygous resistant animals, and will result in populations that are predominantly homozygous resistant.^15^ The objective of this work was to demonstrate that homozygous resistant animals are more tolerant of anticoagulant rodenticides than heterozygous individuals, thus justifying concerns that use of ineffective anticoagulants exacerbates resistance problems and make rodents control progressively more difficult where they exist.

## Conflict of Interest Statement

Stefan Endepols is research and development manager at a company selling several rodenticide products. The other authors are aware of no conflicts of interest.

## Ethical Oversight and Animal Welfare

The study was conducted under a license issued by the UK Home Office and within governance of the Research and Ethics Committee of The University of Reading and under the supervision of a specialist veterinarian.

## Supporting information


**Appendix S1:** Supporting Information

## Data Availability

Research data are not shared.
