# Supplementary material for: Anticoagulant rodenticide blood‐clotting dose‐responses and resistance factors for Tyrosine139Cysteine (Y139C) heterozygous‐ and homozygous‐resistant house mice (Mus musculus)
Source: Pest Manag Sci. 2022 Aug 18;78(11):4480–7. doi: 10.1002/ps.7066 (PMC9804629; doi:10.1002/ps.7066)
Supplement: Supplementary file 1 — Appendix S1: Supporting Information [file PS-78-4480-s001.docx]

Table. S1. Doses of chlorophacinone, diphacinone, warfarin sodium and coumatetralyl administered, numbers of animals dosed and numbers of responders for the susceptible house mouse strain.

| **sex** | **dose rate mg.kg-1** | **number responders** | **number tested** | **% responding** |  | **sex** | **dose rate mg.kg-1** | **number responders** | **number tested** | **% responding** |
| --- | --- | --- | --- | --- | --- | --- | --- | --- | --- | --- |
| **Chlorophacinone** | | | | |  | **Diphacinone** | | | | |
| Female | 0.50 | 0 | 5 | 0.0 |  | Female | 0.50 | 0 | 15 | 0.0 |
|  | 0.60 | 0 | 5 | 0.0 |  |  | 0.80 | 2 | 15 | 13.3 |
|  | 0.70 | 0 | 5 | 0.0 |  |  | 1.00 | 10 | 14 | 71.4 |
|  | 0.75 | 5 | 15 | 33.3 |  |  | 2.00 | 14 | 15 | 93.3 |
|  | 0.80 | 8 | 14 | 57.1 |  | Male | 0.50 | 1 | 15 | 6.7 |
|  | 1.00 | 10 | 15 | 66.7 |  |  | 0.80 | 2 | 15 | 13.3 |
|  | 1.20 | 15 | 15 | 100.0 |  |  | 1.00 | 12 | 15 | 80.0 |
| Male | 0.50 | 0 | 15 | 0.0 |  |  | 2.00 | 15 | 15 | 100.0 |
|  | 0.70 | 0 | 12 | 0.0 |  |  | 3.00 | 8 | 8 | 100.0 |
|  | 0.80 | 12 | 16 | 75.0 |  | **Coumatetralyl** | | | | |
|  | 1.00 | 33 | 44 | 75.0 |  | Female | 1.00 | 0 | 14 | 0.0 |
|  | 2.00 | 32 | 35 | 91.4 |  |  | 2.00 | 0 | 15 | 0.0 |
|  | 3.00 | 6 | 6 | 100.0 |  |  | 3.00 | 4 | 15 | 26.7 |
| **Warfarin sodium** | | | | |  |  | 5.00 | 6 | 15 | 40.0 |
| Female | 0.50 | 1 | 14 | 7.1 |  |  | 6.00 | 7 | 14 | 50.0 |
|  | 1.00 | 2 | 15 | 13.3 |  |  | 7.00 | 12 | 15 | 80.0 |
|  | 2.00 | 11 | 15 | 73.3 |  |  | 8.00 | 10 | 15 | 66.7 |
|  | 2.50 | 9 | 15 | 60.0 |  |  | 9.00 | 14 | 14 | 100.0 |
|  | 3.00 | 15 | 15 | 100.0 |  | Male | 1.00 | 6 | 20 | 30.0 |
| Male | 0.50 | 1 | 15 | 6.7 |  |  | 2.00 | 6 | 15 | 40.0 |
|  | 1.00 | 2 | 15 | 13.3 |  |  | 2.50 | 7 | 15 | 46.7 |
|  | 2.00 | 10 | 15 | 66.7 |  |  | 3.00 | 11 | 16 | 68.8 |
|  | 2.50 | 15 | 15 | 100.0 |  |  | 5.00 | 15 | 15 | 100.0 |

Table. S2. Doses of chlorophacinone, diphacinone, warfarin sodium and coumatetralyl administered, numbers of animals dosed and numbers of responders for the homozygous resistant house mouse strain.

| **sex** | **dose rate mg.kg-1** | **number responders** | **number tested** | **% responding** |  | **sex** | **dose rate mg.kg-1** | **number responders** | **number tested** | **% responding** |
| --- | --- | --- | --- | --- | --- | --- | --- | --- | --- | --- |
| **Chlorophacinone** | | | | |  | **Coumatetralyl** | | | | |
| Female | 0.88 | 0 | 4 | 0.0 |  | Female | 25.00 | 0 | 5 | 0.0 |
|  | 500.00 | 4 | 14 | 28.6 |  |  | 40.00 | 0 | 5 | 0.0 |
| Male | 15.00 | 0 | 3 | 0.0 |  |  | 45.00 | 0 | 19 | 0.0 |
|  | 50.00 | 0 | 3 | 0.0 |  |  | 50.00 | 0 | 10 | 0.0 |
|  | 100.00 | 0 | 4 | 0.0 |  |  | 100.00 | 7 | 28 | 25.0 |
|  | 500.00 | 7 | 15 | 46.7 |  | Male | 20.00 | 2 | 20 | 10.0 |
| **Diphacinone** | | | | |  |  | 25.00 | 7 | 15 | 46.7 |
| Female | 1.24 | 0 | 5 | 0.0 |  |  | 50.00 | 10 | 22 | 45.5 |
|  | 500.00 | 6 | 14 | 42.9 |  |  | 100.00 | 19 | 34 | 55.9 |
| Male | 15.00 | 0 | 3 | 0.0 |  | **Warfarin sodium** | | | | |
|  | 50.00 | 0 | 4 | 0.0 |  | Female | 300.00 | 1 | 15 | 6.7 |
|  | 100.00 | 0 | 4 | 0.0 |  |  | 400.00 | 2 | 16 | 12.5 |
|  | 500.00 | 10 | 16 | 62.5 |  | Male | 200.00 | 1 | 16 | 6.3 |
|  |  |  |  |  |  |  | 280.00 | 1 | 15 | 6.7 |
|  |  |  |  |  |  |  | 300.00 | 10 | 15 | 66.7 |
|  |  |  |  |  |  |  | 500.00 | 0 | 2 | 0.0 |

Table. S3. Doses of brodifacoum, bromadiolone, difenacoum, difethialone and flocoumafen administered, numbers of animals dosed and numbers of responders for the heterozygous resistant Y139C house mouse strain.

| **Sex** | **Dose rate (mg kg^-1^)** | **Number of responders** | **Number tested** | **% responding** |  | **Sex** | **Dose rate (mg kg^-1^)** | **Number of responders** | **Number tested** | **% responding** |
| --- | --- | --- | --- | --- | --- | --- | --- | --- | --- | --- |
| **Brodifacoum** | | | | |  | **Difethialone** | | | | |
| Female | 0.45 | 0 | 11 | 0.0 |  | Female | 0.40 | 0 | 11 | 0.0 |
|  | 0.60 | 7 | 16 | 43.8 |  |  | 0.40 | 0 | 20 | 0.0 |
|  | 0.85 | 7 | 10 | 70.0 |  |  | 0.75 | 2 | 6 | 33.3 |
| Male | 0.55 | 8 | 24 | 33.3 |  |  | 1.00 | 12 | 25 | 48.0 |
|  | 0.75 | 18 | 34 | 52.9 |  |  | 1.20 | 4 | 16 | 25.0 |
|  | 0.90 | 17 | 20 | 85.0 |  | Male | 0.40 | 0 | 15 | 0.0 |
| **Bromadiolone** | | | | |  |  | 0.50 | 3 | 20 | 15.0 |
| Female | 25.00 | 2 | 16 | 12.5 |  |  | 1.00 | 5 | 15 | 33.3 |
|  | 35.00 | 8 | 51 | 15.7 |  |  | 1.20 | 4 | 8 | 50.0 |
|  | 60.00 | 14 | 15 | 93.3 |  | **Flocoumafen** | | | | |
|  | 100.00 | 5 | 5 | 100.0 |  | Female | 0.25 | 0 | 15 | 0.0 |
| Male | 15.00 | 2 | 14 | 14.3 |  |  | 0.30 | 0 | 15 | 0.0 |
|  | 25.00 | 12 | 24 | 50.0 |  |  | 0.40 | 0 | 16 | 0.0 |
|  | 60.00 | 18 | 24 | 75.0 |  |  | 0.45 | 3 | 13 | 23.1 |
|  | 100.00 | 22 | 29 | 75.9 |  |  | 0.50 | 17 | 26 | 65.4 |
| **Difenacoum** | | | | |  |  | 0.65 | 7 | 10 | 70.0 |
| Female | 1.00 | 0 | 5 | 0.0 |  |  | 1.00 | 17 | 17 | 100.0 |
|  | 1.30 | 5 | 16 | 31.3 |  | Male | 0.25 | 0 | 5 | 0.0 |
|  | 1.35 | 5 | 16 | 31.3 |  |  | 0.30 | 2 | 5 | 40.0 |
|  | 1.40 | 8 | 16 | 50.0 |  |  | 0.40 | 6 | 15 | 40.0 |
|  | 1.50 | 9 | 15 | 60.0 |  |  | 0.45 | 8 | 15 | 53.3 |
|  | 2.50 | 4 | 5 | 80.0 |  |  | 0.50 | 10 | 13 | 76.9 |
|  | 5.00 | 5 | 5 | 100.0 |  |  | 0.70 | 5 | 10 | 50.0 |
| Male | 0.50 | 0 | 14 | 0.0 |  |  | 1.00 | 9 | 9 | 100.0 |
|  | 0.80 | 2 | 6 | 33.3 |  |  |  |  |  |  |
|  | 1.00 | 6 | 21 | 28.6 |  |  |  |  |  |  |
|  | 1.10 | 9 | 15 | 60.0 |  |  |  |  |  |  |
|  | 1.20 | 9 | 10 | 90.0 |  |  |  |  |  |  |
|  | 1.30 | 6 | 9 | 66.7 |  |  |  |  |  |  |
|  | 1.40 | 5 | 5 | 100.0 |  |  |  |  |  |  |
|  | 1.50 | 4 | 5 | 80.0 |  |  |  |  |  |  |
